# Functional Genomics of Brain Aging and Alzheimer’s Disease: Focus on Selective Neuronal Vulnerability

**DOI:** 10.2174/138920210793360943

**Published:** 2010-12

**Authors:** Xinkun Wang, Mary L Michaelis, Elias K Michaelis

**Affiliations:** Higuchi Biosciences Center and Department of Pharmacology and Toxicology, The University of Kansas, Lawrence, KS 66047, USA

**Keywords:** Selective neuronal vulnerability, aging, Alzheimer’s disease, functional genomics, neuroinflammation, energy metabolism, synaptic neurotransmission.

## Abstract

Pivotal brain functions, such as neurotransmission, cognition, and memory, decline with advancing age and, especially, in neurodegenerative conditions associated with aging, such as Alzheimer’s disease (AD). Yet, deterioration in structure and function of the nervous system during aging or in AD is not uniform throughout the brain. Selective neuronal vulnerability (SNV) is a general but sometimes overlooked characteristic of brain aging and AD. There is little known at the molecular level to account for the phenomenon of SNV. Functional genomic analyses, through unbiased whole genome expression studies, could lead to new insights into a complex process such as SNV. Genomic data generated using both human brain tissue and brains from animal models of aging and AD were analyzed in this review. Convergent trends that have emerged from these data sets were considered in identifying possible molecular and cellular pathways involved in SNV. It appears that during normal brain aging and in AD, neurons vulnerable to injury or cell death are characterized by significant decreases in the expression of genes related to mitochondrial metabolism and energy production. In AD, vulnerable neurons also exhibit down-regulation of genes related to synaptic neurotransmission and vesicular transport, cytoskeletal structure and function, and neurotrophic factor activity. A prominent category of genes that are up-regulated in AD are those related to inflammatory response and some components of calcium signaling. These genomic differences between sensitive and resistant neurons can now be used to explore the molecular underpinnings of previously suggested mechanisms of cell injury in aging and AD.

## 1 INTRODUCTION

Brain aging and associated neurodegenerative diseases such as Alzheimer’s disease (AD), do not affect all neurons equally. For example, in the hippocampus, neurons in the CA1 region are vulnerable to brain aging and AD, but those in the nearby CA3 region are not nearly as heavily damaged as the CA1 neurons [1-3]. A pattern of selective loss of synapses and neurons in certain brain regions has been described for both the aging process [4-6] and AD [7-10]. These studies have been performed, for the most part, at the microanatomical level and have identified relatively few neurochemical changes that correlate with either neuronal vulnerability or resistance to age- or AD-associated injury or death. The selective vulnerability of certain brain neurons appears to be an intrinsic characteristic of these neurons. Besides aging and AD, this phenomenon, of selective neuronal vulnerability (SNV), is also a characteristic of many other neural insults, such as Parkinson’s disease [11], amyotrophic lateral sclerosis (ALS) [12], ischemia [13], epileptic seizures [14], and oxidative stress (OS) [15, 16]. Yet, SNV is often overlooked in the study of brain aging and neurodegenerative diseases. By definition, SNV refers to the fact that, only select populations of neurons are uniquely vulnerable to injury or death under adverse conditions, whereas other neurons are relatively resistant to such stresses in their environment. The selective vulnerability of some neurons is often manifested in structural and functional changes that may or may not lead to the death of the cells. For example, vulnerable neurons often suffer loss of dendrites that leads to a significant impairment of synaptic transmission, but the cells may still survive for a time in this altered state. Understanding the mechanisms underlying SNV is an essential step in efforts to develop strategies to moderate the deleterious impact of aging and neurodegenerative diseases on the overall quality of life. 

The aging process and age-associated disease conditions, including AD, are marked by genomic instability and consequential or compensatory changes in gene expression patterns [17-19]. Since the functional status of cells is determined to a large extent by their genomic activity, genomic studies of neurons that are selectively vulnerable to brain aging and AD, are expected to yield new insights into the intrinsic biochemical and cell biological processes that make some neurons susceptible to a wide variety of stresses. In this review, we first describe the brain regions and/or neuronal populations that are currently known to be most affected by aging and AD. Secondly, we collect and carry out an analysis of the published functional genomic studies on the phenomenon of SNV in brain aging and AD. Finally, we attempt to integrate and discuss the findings from multiple studies into some common patterns or characteristics that seem to offer the most likely explanations for the differential vulnerability of neuronal populations to stresses due to both aging and to disease.

## 2 SELECTIVE NEURONAL VULNERABILITY IN BRAIN AGING

### 2.1 Brain Regions that are Vulnerable to Normal Aging

As indicated above, several brain regions uniquely susceptible to age-dependent cell damage ultimately disrupt normal function and compromise behavioral performance. The frontal cortex is one such region that plays a pivotal role in cognition and memory, and even subtle changes in the neuronal environment of selectively vulnerable neurons appear to lead to the cognitive impairment characteristic of normal brain aging [20, 21]. In efforts to identify the most age-sensitive regions in the brain, non-invasive techniques such as structural brain imaging and functional magnetic resonance imaging are being widely used and combined with knowledge derived from post-mortem analyses of aging human brain. These studies, based mostly on volumetric measurements in human brain, show that the association cortex, the neostriatum, and the cerebellum are the most vulnerable regions to age-dependent loss of volume, whereas the primary sensory cortices (such as the visual cortex), the entorhinal cortex, the paleostriatum, and the pons show much less shrinkage [22, 23]. (See Fig. (**1**) for location of several of these brain regions). 

It is important to note that there are variable patterns in different brain regions with regard to volumetric changes during aging, or even during development. For example, the cerebellum does not change in volume from middle to old age. However, significant cerebellar volume decreases occur between young adulthood and middle age, with much less change taking place between middle and old age. Thus the volume shrinkage of the cerebellum occurs early in life and then slows down in the mid-50’s in humans [24]. In addition, it has been observed that during development, some cortical regions exhibit a continuous increase in volume, whereas in the frontal and parietal cortices the increase is followed by a decrease in volume during the transition from adolescence to young adulthood [25]. These changes in brain structure are not accompanied, of course, by cognitive decline in young individuals thus revealing the complexity faced with trying to link brain imaging changes and the cause of alterations in neurological function. Other indices of brain activity besides volume changes, such as positron emission tomography (PET) of cerebral metabolic rate of glucose (CMR_Gl_), might provide additional correlative measures of structure to function in the human brain.

The brain volumetric measurements as well as the PET measures of CMR_Gl_ reinforce the notion that aging affects some regions of the brain more than others. In addition to the volume changes in select brain regions of the brain during aging, it has been repeatedly shown that the frontal cortex shows the greatest and most consistent decrements in CMR_Gl_ as compared with all other regions in the cortex or subcortical components of the aging brain [26, 27]. These changes in metabolic activity in select cortical areas during aging are either related to altered neuronal expression of some key enzymes controlling the overall metabolic state of neurons and associated glial cells, or they are the result of altered activation of synapses or of the disruptive effects of abnormal neuronal excitability during aging. Gene expression analyses, in combination with neuro-imaging studies (including PET scan), as well as detailed microanatomical investigations, are providing new windows onto the molecular and cellular changes that might account for such differential patterns of neuronal susceptibility to the aging process and to age-related diseases. It should be emphasized, however, that neuronal losses during aging even in select, sensitive regions are relatively modest, whereas decreases in the number of synapses in the same regions appear to be a more prominent characteristic of brain aging. These observations have led to the assertion that most of the functional decline associated with normal aging is caused by relatively subtle changes, such as loss of dendrites, reductions in spine densities, altered spine morphologies, or changes in the molecular profile of synapses [21, 28-31]. 

### 2.2 Functional Genomic Studies on Brain Regions Most Vulnerable to Aging 

#### 2.2.1 Human Studies

In order to study how brain regions differentially respond to the stresses associated with increasing age, some investigators have used functional genomics approaches, though the number of these studies is still rather limited. Nevertheless, such studies have provided further support for the concept of regional heterogeneity with respect to the differential rates of aging-associated changes. An initial report of differential gene expression patterns in three human brain regions was that by Evans *et al.* [32] who examined gene expression in cerebellar cortex and two cerebral cortical regions (anterior cingulate cortex and dorsolateral prefrontal cortex). The microarray gene expression patterns show that the two regions of the cerebral cortex had similar levels of expression of most genes and that these two cortical regions differed significantly in terms of gene expression patterns from those of the cerebellar cortex [32]. More than one thousand transcripts were differentially expressed in the cortical vs. cerebellar regions and the most prominent ontological categories among the differentially expressed genes were those of signal transduction, neurogenesis, synaptic transmission, and transcription factor regulation. In another study of regional differences in gene expression in human brain, Khaitovich *et al.* [33] analyzed patterns of expression in six areas of the brain: cerebellum, caudate nucleus, dorsolateral prefrontal cortex, anterior cingulate cortex, primary visual cortex, and Broca’s area. Consistent with the study by Evans *et al.* [32], Khaitovich *et al.* also found that while the four regions of the cerebral cortex are similar to each other, the overall transcriptomic profiles of the cerebral cortex, the caudate nucleus and the cerebellum differ significantly from each other [33]. The gene ontology categories that differed most in terms of expression in the various brain regions were those of synaptic transmission, signal transduction, neurogenesis, neuronal development, and calcium ion [Ca^2+^] regulation. Because the analyses of gene expression patterns in both the Evans *et al.* and Khaitovich *et al.* studies contained too few brain samples across the aging spectrum (45 to 88 years of age), Fraser *et al.* [34] conducted a meta-analysis on the results of these two studies using the aging-related pattern of changes in gene expression in the frontal pole of the human brain identified in the study by Lu *et al.* [18]. Using the 841 genes identified as showing a pattern of either increasing or decreasing expression with advancing age in the Lu *et al.* study, they calculated the correlation coefficients resulting from comparisons of aging-related profiles between two tissues (Spearman rank correlation, r, and significance P values), the frontal pole of the Lu *et al.* study and one of the tissues studied by Khaitovich *et al.* The investigators described a highly significant correlation between the frontal pole in the Lu *et al.* study and each of the four regions of the cerebral cortex in the Khaitovich *et al.* study (anterior cingulate cortex, Broca’s area, prefrontal cortex, and primary visual cortex, r>0.8 and p<0.02), but a lack of correlation between the frontal pole and the two non-cerebral-cortical regions (caudate nucleus and cerebellum, r<0.1 and p>0.4) in the Khaitovich *et al.* study. The meta-analysis confirmed the heterogeneity in age-dependent genome-wide gene expression profiles across different brain regions and demonstrated that the expression patterns of the five cerebral cortical regions are quite similar to each other in terms of the rates of appearance of age-related changes, whereas the cerebellum and caudate nucleus respond to aging very differently. Further analyses of the difference between the cerebral cortical regions and cerebellum revealed that the number of genes whose expression pattern changes with advancing age is lowest in the cerebellum. 

A more recent study traced gene expression changes in four regions of postmortem brains in 55 normal individuals aged 20-99 years [35]. Similar to the studies described above, GeneChip data from this study again showed the regional heterogeneity in gene expression patterns in human brain during the aging process. However, unlike previous studies that reported significantly higher numbers of genes whose expression was changing with advancing age in vulnerable brain regions as compared with less vulnerable regions, this study indicated that two regions that are vulnerable to aging and AD, the hippocampus and the entorhinal cortex, displayed fewer aging-responsive genes than the superior frontal gyrus and post-central gyrus, regions that are less vulnerable to age-associated neuronal loss. It is important to note, however, that the relative differences in age-related neuronal injury or loss in these neocortical and paleocortical regions are less pronounced than the differences between, for example, cerebral, cortical and cerebellar regions. Therefore, the results of the study by Berchtold *et al.* [35] may have identified more subtle differences among generally vulnerable brain regions. 

Principal component analysis of gene expression in single neurons captured by laser capture microdissection (LCM) from the two vulnerable regions of hippocampus and entorhinal cortex revealed that these neurons expressed 102 genes at lower levels than did neurons in regions that are less susceptible to the aging process, such as the superior frontal gyrus and posterior cingulated cortex [36]. Beyond the fact that the results from this study confirm the differential pattern of gene expression across several brain regions, they also point to some specific genes whose relatively low level of expression in susceptible regions in the aged brain (mean age: 79.8 ± 9.1 years) may predispose these neurons to differential vulnerability to age and AD-associated injury and death. Among the top categories of genes that were under-expressed in neuronal populations in the hippocampus and entorhinal cortex were those related to Ca^2+^ homeostasis, such as *Slc8A1* (solute carrier family 8—sodium/calcium exchanger—member 1), *Ppp2ca* (protein phosphatase 2, catalytic subunit, α isoform) and *Calm2* (calmodulin 2), and those associated with intracellular signaling.

#### 2.2.2 Animal Studies

As described above, the aging process is associated with specific gene expression patterns in the human brain but the genes expressed differentially in other species during the aging process differ from those detected in human brain even among close relatives of humans, such as the chimpanzee [34]. Despite the differences in specific gene expression patterns among various species, neurons in certain brain regions, such as the hippocampus CA1 field and the basal forebrain cholinergic neuronal region, are selectively vulnerable in all species [16, 37-41]. Using a rodent model of brain aging, Xu *et al.* [42] studied five central nervous system regions (cerebral cortex, hippocampus, striatum, cerebellum and spinal cord) across three age groups, 6 (young), 16 (middle age), and 24 (old) month old animals. Each of these regions was shown to have a unique transcriptomic signature. Spinal cord has the most genes whose expression changes as a result of aging (600+), the cerebral cortex and hippocampus are next (each with 400+ genes), while the striatum and cerebellum have the fewest age-related changes in gene expression (with only 100+ differential genes). Although these gene expression patterns differ somewhat from those detected in the human brain, they nevertheless were consistent with the idea of heterogeneity in brain aging across different regions, and with the notion that certain regions, such as the cerebellum, exhibit the lowest levels of change in gene expression related to the aging process. Therefore, patterns of gene expression in other species besides humans may provide additional information about specific molecular changes that are related to the vulnerability of some cells to the aging process, even though the specific genes affected by aging may differ among animal species. 

Functional categorization of aging-associated gene expression changes in rodent brain indicated that all regions show decreases in the expression of genes related to mitochondrial function with the decreases being more significant in the cerebral cortex, hippocampus, and spinal cord [42]. The transcriptomic data also match fairly well with the observed pattern of decreases with age in mitochondrial metabolic capacity of small to medium-sized mitochondria in human cerebellum [43]. The mitochondrial metabolic capacity decreases from young to middle age but stays stable from middle to old age. The transcriptomic studies in mice and the mitochondrial metabolism studies in human cerebellum might indicate that this region of the brain is relatively plastic with respect to regulation of gene transcription and metabolism before middle age but becomes relatively insensitive, transcriptionally and metabolically, to the aging process after middle age. The differential response of cerebellar neurons at middle age and old age might be due to the lower metabolic rate of cerebellar neurons as has been observed for both human and primate brains [44-46]. An associated effect derived from the low levels of mitochondrial metabolic activity in aging cerebellum would be a lowering of the levels of reactive oxygen species (ROS) and OS, as well as of DNA oxidative damage that results from elevations in ROS levels in neurons [47, 48]. Decreased levels of oxidatively modified DNA would protect cerebellar neurons from damage that might otherwise occur in this brain region. Thus, the study of gene expression patterns in animal and human brain during aging has enhanced our understanding of the changes that occur at the genomic level and which might identify the molecular causes for the age-associated structural, physiological and biochemical changes. 

## 3 SELECTIVE NEURONAL VULNERABILITY IN ALZHEIMER’S DISEASE

### 3.1 Brain Regions that are Vulnerable to Alzheimer’s Disease

AD is characterized by the accumulation of oligomeric or aggregated amyloid β peptides (Aβ) in senile plaques (SPs), fibrils of hyperphosphorylated tau protein in neurofibrillary tangles (NFTs), and more importantly, by the loss or injury of neurons in select brain regions [49]. The cerebral cortex, especially the association areas, and the hippocampus, are the predominant regions of AD pathology [4, 50, 51]. Among these regions, the most vulnerable neurons are those in layer II of the entorhinal cortex, the subiculum, and the CA1 region of the hippocampus; these neuronal populations consistently display high levels of neurofibrillary tangles (NFTs) and are first lost in the early phases of the disease [52-54]. With regard to the distribution of NFTs across the different regions of the cerebral cortex, neurons of the temporal cortex contain more NFTs than those (in decreasing order) of the frontal, parietal, occipital, and posterior cingulate cortex [55, 56]. Besides the neurons in the cerebral cortex and the hippocampus, many subcortical structures that have connections to the cerebral cortex also contain neurons with large amounts of NFTs, including the amygdala, nucleus basalis of Meynert, ventral tegmental area, dorsal raphe, locus coeruleus, olfactory bulb, and some midline thalamic and hypothalamic nuclei [57, 58]. In contrast to neurons in these regions that contain high levels of NFT’s, primary sensory and motor neurons are largely spared in the disease [59, 60]. 

Among the most vulnerable neurons in AD are the large pyramidal neurons in the areas mentioned above, particularly association neurons with long projections [61, 62]. Examples of such neurons are those in the entorhinal cortex layer II which provide the major cortical input to the hippocampus through the perforant pathway, and those in the subiculum which represent the major output of the hippocampus to the prefrontal cortex and other brain regions [63]. The underlying factors for the vulnerability of these large-sized neurons are thought to be their high demand for energy that leads to high levels of oxidative phosphorylation. Oxidative phosphorylation depends on the activity of the electron transport chain (ETC) enzymes, and hyperactivation of these enzymes is known to lead to OS and may thus bring about protein, DNA, and lipid modifications that are injurious to these neurons. In addition, the relatively large surface area of these neurons may increase exposure to extracellular toxic agents while their long processes would put increased demands on axoplasmic transport over long distances. Finally, neurons with long processes also have high levels of neurofilaments whose protein subunits tend to form aggregates during cellular stress.

### 3.2 Functional Genomic Studies on Selective Neuronal Vulnerability in Alzheimer’s Disease

Several investigators have explored the mechanisms of region-specific neurodegeneration in AD by performing functional genomic studies on the most vulnerable brain regions affected by the disease. A list of these studies (up to October 2010) is presented in Table **1** for human subjects and Table **2** for animal models of AD. The studies summarized in those two tables vary in their experimental approaches and include transcriptomic studies of individual regions of the brain as well as cross-sectional studies comparing regions that exhibit differential neuronal vulnerabilities. As will be noted, some of the studies are based on an analysis of gene expression patterns in a whole brain region, whereas others represent high-resolution analyses of gene expression patterns in individual neurons within a given region. Despite these different experimental approaches, several common patterns are apparent, and these commonalities provide some mechanistic insights into the question why only certain regions of the brain or certain neuronal populations are most vulnerable in AD. 

#### 3.2.1 Gene Expression Patterns in Different Brain Regions in AD

The region-specific studies cover most brain areas affected by AD, including the entorhinal cortex, hippocampus, temporal cortex, frontal cortex, parietal cortex, cingulate cortex, amygdala, and nucleus basalis of Meynert. Because of their significance in cognition and memory formation, gene expression patterns in the hippocampus and frontal cortex have been examined in several of these studies. An effective approach to study SNV in AD is to compare vulnerable regions with resistant regions. As listed on the top of Table **1**, a number of vulnerable vs. resistant regional comparisons were conducted, covering the majority of the vulnerable regions listed above. Regions of the brain populated with neurons that are resistant to AD-related damage included the striatum, visual cortex, cerebellum, and brainstem. However, many of the existing genomic expression studies shown in Table **1** were not direct, side-by-side comparisons of the patterns of gene expression in vulnerable vs. resistant regions. For many of these studies, only vulnerable regions were used and the corresponding analyses were focused on how the patterns observed in these vulnerable regions differed between brains from AD subjects and those from age-matched controls. Although the majority of the gene analyses were carried out with RNA isolated from dissected brain regions, those described in references 46 and 47 were done with RNA isolated from cells selected by LCM.

#### 3.2.2 Methodological Considerations with Regard to Gene Expression Studies in AD

It is well known that within a brain region characterized by overall sensitivity to stress there are cells that are resistant to the stress stimuli. A good example of this is the high level of sensitivity to OS, ischemia-induced injury, or glutamate toxicity of pyramidal neurons in the CA1 subfield of the hippocampus whereas the proximal neurons in the CA3 subfield are resistant to the same adverse conditions [64-67]. With regard to AD-associated neuronal injury or loss, the hippocampus as a whole is usually considered as a vulnerable region but the CA1 pyramidal neurons are much more susceptible to the pathogenic condition in AD than are neurons in the CA3 subfield or in the dentate gyrus [8]. Therefore, studies of brain metabolism and or gene expression that examine regional brain differences are actually reporting on the average metabolic or transcriptomic activity of a region that is composed of both sensitive and resistant neurons. 

Although it is ideal to conduct studies on SNV using only vulnerable neurons rather than all of the cells in a given region, the technical difficulties involved in collecting individual neurons still poses a considerable challenge to investigators. For this reason, most published genomic expression studies of SNV in AD are studies of whole brain regions. In such studies, unique markers of vulnerable neurons might be masked by signals from stress-resistant neurons, and differences between vulnerable and resistant neurons can be diluted by background noise from other cells in a given brain region, such as glial cells. But, at the single cell level, the ratio of genuine signals to false signals or background noise is much greater making the results more dependable. Recent developments in selective cell procurement technologies, such as LCM, have made high-resolution analyses a reality [68]. The low mRNA yield that accompanies the usually small number of cells captured by LCM can be remedied by linear amplification of the messenger molecules [69]. As shown in Table **1**, among existing studies, several groups employed the target cell capture approach and demonstrated the feasibility and power of gene expression analysis performed on a uniform population of cells [70-72]. 

The vast majority of functional genomic studies related to regional vulnerability in AD have used postmortem brain tissue from human AD subjects and the respective age-matched controls (Table **1**). In most of these studies, fewer than 10 human brains per group were analyzed. The largest population of AD cases reported thus far involved analysis of samples from 61 AD patients [73]. In this review, we have also examined the patterns of gene expression in experimental animals that are used as surrogates of human AD so that we may determine whether animals thought to represent models of AD exhibit similar patterns in terms of selectively vulnerable or resistant neuronal populations as do humans. The animal models used are, for the most part, transgenic mice that over-express one to three genes associated with familial cases of AD. These are the transgenic mice for amyloid precursor protein with Swedish mutation (APPsw) and those with APP plus presenilin 1 (PS1), as well as the double gene knockout mice for PS1 plus PS2. In addition, brain tissue from wild type mice injected with β-amyloid has been analyzed in order to determine the gene expression patterns in vulnerable vs. resistant neuronal populations. The findings from these animal studies are summarized in Table **2** and the observed gene expression patterns integrated into the overall pattern of SNV in AD. 

Early transcriptomic studies of AD-vulnerable regions focused mainly on the identification of differentially expressed genes that investigators believed to be important for the disease process. Although attention to single gene changes may provide valuable information about potential targets for therapeutic intervention in AD, such an approach does not allow for the visualization of possible gene interactions and the effect that such interactions may have on disease-relevant molecular pathways in cells. To achieve an understanding of the disease process, a “systems approach” to analyzing transcriptomic data should provide a more comprehensive view of the disease process [74] and would, therefore, be the preferable approach for data analysis in such studies. Bioinformatic analyses of transcriptomic data now encompass the identification of genes, gene ontologies, bio-functions, biological pathways, and gene networks. More recent studies of SNV in AD brains have made use of gene ontological annotations, expert annotated and compiled bio-functions (such as those offered by Ingenuity Systems), and the currently known biological pathways, in order to identify key gene ontologies, bio-functions and pathways that can differentiate vulnerable from resistant neurons in AD. These higher-level analyses have provided more insight into the underlying mechanisms of SNV in AD than the early studies did. 

#### 3.2.3 Possible Common Mechanistic Factors Determining Neuronal Vulnerability in AD

It is usually not easy to determine whether the findings from a single transcriptomic study can be used in deriving general principles about the state of neuronal injury in the brain. Added to this uncertainty are possibly confounding experimental parameters such as brain region, cellular resolution, brain tissue origin, and others already mentioned above. In an effort to reach the goal of understanding the key mechanistic factors underlying SNV in AD, we have collected data from existing studies and have attempted to determine if there are common mechanistic factors that have been identified by many of the studies we have examined. Shared key bio-functions and pathways identified in the studies we have summarized were used to arrive at possible mechanisms for differential vulnerability of neurons in AD. The factors we have identified represent the most prominent characteristics of AD-affected regions and are factors that were reproducibly observed in many studies, despite the use of different regions or diverse neuronal populations from the brains of either human subjects or animal models. The key biological functions and molecular pathways that seem to distinguish vulnerable from resistant neurons in AD are outlined below, and some of the key genes in these functions are presented in Table **3**.

##### a Decreases in Mitochondrial Metabolism and Energy Production

Since the metabolic activity of the brain is very high, accounting for an estimated 20-25% of glucose and oxygen utilization in the body, it is not surprising that reduced metabolism and energy production might be significantly correlated with cognitive decline in AD [75, 76]. The CMR_Gl_ is significantly lower in AD-affected brain regions when compared with the same brain regions of age-matched healthy controls [77-80]. On the other hand, the same measurements of the metabolic rate in regions spared by the disease process are equivalent to those in the brains of control subjects. Decreases in CMR_Gl_ in AD have been reported for regions that are most vulnerable as defined by neuropathological changes, including those of the frontal, parietal, temporal, and posterior cingulate cortex, as well as the hippocampus and entorhinal cortex [80-82]. It is also highly significant that the regions that tend to accumulate the highest amounts of Aβ in AD, or in cognitively normal individuals with amyloid plaques in their brain, differ from other brain regions in terms of the overall levels of glycolytic activity [83, 84]. Glucose metabolism in brain is generally through the pathway that includes oxidative phosphorylation in mitochondria but, apparently, in the most vulnerable regions of the brain, glucose metabolism is through the glycolytic pathway, a pathway that leads to more rapid utilization of glucose but less ATP generation. Thus, differences in energy metabolism and glucose utilization in brain are emerging as prominent features of an AD brain as compared with non-AD brains. As shown in Tables **1** and **2**, the down-regulation of genes related to metabolic homeostasis and energy production is a recurring theme in AD brains [71, 72, 85-90]. For example, Liang *et al.* [72] compared expression of 80 metabolically relevant genes in brain regions differentially affected by AD. Compared with controls, vulnerable regions in brains of AD cases exhibited significant decreases in the expression of many genes related to cellular metabolism. In the posterior cingulate cortex, 70% of the nuclear genes that encode mitochondrial ETC subunits were expressed at significantly lower levels in the AD cases compared with controls. In middle temporal gyrus and hippocampal CA1 subfield, expression levels of 61% to 65% of these genes were significantly lower in AD brains *vs.* those in age-matched controls. In comparison, in a region that is relatively spared by AD, i.e., the visual cortex, only 16% of the genes were expressed at lower levels than in controls. This detailed study, along with many others, demonstrates that there is a decline in mitochondrial energy metabolism in the AD brain, and that the regions that suffer the greatest decline in genes related to cell metabolism and energy production are those that are most vulnerable to the disease process in AD. Among the mitochondrial ETC proteins whose genes are down-regulated in AD, the genes *Cox4* (Cytochrome c oxidase, subunit IV) and *Uqcrc2* (Cytochrome bc-1 complex core protein) are the most consistently down-regulated genes. It is important to point out that in addition to the lower expression levels of genes related to ETC enzymes in vulnerable neurons in the AD brain, those for subunits of the translocases of the outer and inner mitochondrial membranes (*Tomm* and *Timm*) are also down-regulated in these regions [91]. These translocases play an important role in the import of mitochondria-targeted proteins from the cytoplasm through the outer and inner mitochondrial membrane. Included in the group of proteins transported into mitochondria are many of the ETC enzymes. To add to the importance of TOMM proteins in AD, new polymorphisms associated with AD (multiple T bases) have now been identified in an intron of the gene for one of the subunits of the TOMM complex, *Tomm40* [92]. This genetic variation leads to the development of late onset AD at an earlier age than most such cases of late onset AD.

##### b Increases in Gene Expression of Inflammatory Response Genes

In many of the studies listed in Table **1** [85, 87, 90, 93-98], inflammatory responses are among the most pronounced bio-functions or pathways associated with up-regulated genes in affected AD neurons. Aβ aggregation and deposition in AD brains is known to be neurotoxic and to elicit the production of cytokines, chemokines, and other neuroinflammatory mediators from microglia, the immune system cells of the brain [99, 100]. Up-regulated genes involved in this response, as uncovered by functional genomic studies, include those for: 1) cytokines (such as interleukin[IL]-1α, IL-1β, IL1F7, interferon α5, interferon γ, interferon λ2, transforming growth factors β1 and β3, and tumor necrosis factor α); 2) chemokines (such as CCL5 and CCL27); 3) cytokine/chemokine receptors (such as chemokine C-X-C motif receptor 2 [CXCR2], CXCR4, CCR3, IL-1 receptor type I, IL-2 receptor γ, IL-10 receptor α, IL-10 receptor β, IL-13 receptor α1, and receptors of IL-6, IL-17, and IL-18); 4) proinflammatory transcription factors (such as NF-IL6 and NF-κB 2) and other pro-inflammatory proteins (such as interferon-induced protein 3 or IFITM3). Not all of these up-regulated genes (or their products) are pro-inflammatory, of course, since some of the gene products, such as those of the IL-10 receptor, are anti-inflammatory [101]. Overall, these findings are consistent with other experimental evidence showing that in AD brains, inflammation is particularly localized in regions most affected by the disease [102-109]. The up-regulation of some anti-inflammatory genes may be indicative of a compensatory response to inflammation in AD brains. Based on the fact that increased expression of inflammatory response genes is primarily focused on the AD-vulnerable regions, it is highly likely that neuroinflammation is a significant contributor to the pathogenic cascade that is activated in AD [110]. Although the activation of inflammatory responses in AD-affected regions might be neuroprotective at the early stage of the disease by controlling and minimizing the damaging effects of Aβ, chronic inflammation and long exposure to inflammatory response effectors might well be detrimental to neurons and glial cells in later stages of the disease.

##### c Dysfunction of Synaptic Transmission and Synaptic Vesicle Transport

The third common transcriptional profile identified in many of the studies is the alteration in levels of gene expression related to synaptic activity in vulnerable neurons [70-72, 98, 111-113]. Since synaptic activity is fundamental to memory formation and cognitive function, the region-specific decreases in gene expression related to synaptic neurotransmission uncovered in these studies can be viewed as a neurobiological correlate of AD-associated cognitive decline. Specifically, these studies demonstrate that the expression of many important genes for synaptic transmission or vesicular transport are significantly down-regulated, including the genes for neuritin, synapsin I, synapsin II, synaptobrevin, synaptojanin, synaptophysin, synaptopodin, synaptotagmin, syntaxin, α-synuclein, and β-synuclein. The down-regulation in sensitive regions of genes related to synaptic vesicle docking and recycling at the synapse, such as dynamin I, is also likely to be linked to the decline in cognitive function in AD, because of the ensuing decreases in neurotransmitter release at synapses. This observation suggests a selective weakening or loss of neuronal connections in AD-affected brain regions, and may help explain the decreases in synaptic density observed by quantitative morphometric analyses performed on brain regions vulnerable to AD-induced damage [114-116]. Biochemical analyses of these protein products are in agreement with the transcriptional profile described above [117-119]. Region-specific dysfunction in synaptic transmission may result not only from the down-regulation of the genes described above, but also from direct effects of Aβ on synaptic macromolecules [120, 121], or from the effects of products of chronic inflammatory response, such as tumor necrosis factor [122], or from other factors some of which are discussed below. 

##### d Calcium Regulation, Cytoskeletal Function, Signal Transduction, and Proteolytic Activity

Additional transcriptomic alterations that might also play a role in AD-associated SNV were identified in the functional genomic studies. These include: 1) Up-regulation in AD-vulnerable regions of genes related to calcium (Ca^2+^) homeostasis and signaling pathways, such as the inositol trisphosphate (IP_3_)-receptor kinase and the Ca^2+^-regulated phosphatase calcineurin (*Ppp3c*), as well as the down-regulation of genes encoding calcium binding proteins [73, 85, 86, 95, 123]; 2) Down-regulation of the expression of genes for cytoskeletal proteins, e.g., β-tubulin and neurofilament subunits *Nf-L, Nf-M, Nf-H* and *Nf-66*, as well as those for some axonal cargo transporters in AD brains [70, 85, 90]; 3) Down-regulation in AD brains of genes involved in signal transduction pathways, such as G-protein-coupled receptor (GPCR) signaling [85, 90, 124]; 4) Down-regulation of genes for neurotrophic factors and their receptors, such as *Bdnf* (brain-derived neurotrophic factor) and the receptors *TrkB* and *TrkC* [87, 97, 113]; and 5) Up-regulation of the genes for cathepsin D, H, S and Y, a family of endolysosomal proteases involved in the proteolytic cleavage of APP and the formation of Aβ [70, 87, 93, 95, 113]. 

## 4 CONCLUDING REMARKS

In the preceding sections we have presented information from microanatomical, brain imaging, functional imaging, and functional genomic studies in order to begin to define the intrinsic characteristics of selectively vulnerable neurons in the aging brain and in AD. Potential candidate neurochemical processes have been suggested in the past as possible determinants of differential vulnerability, especially the expression of high levels of neurofilament proteins in vulnerable neurons and of calcium-binding proteins in resistant neurons [6]. However, the information gleaned from those studies, although accurate, was relatively limited when compared with the large data sets available to us today as a result of whole genome expression studies. The key functional gene categories that have emerged from our review of studies that distinguish vulnerable from resistant neurons are summarized in Fig. (**2**). The summary shown in Fig. (**2**) highlights what appear to be intrinsic differences between vulnerable and resistant neurons in terms of gene expression, regardless of the site of their location in brain. 

The summary of studies at the genomic level presented in this review, points to some commonalities between aging and AD, the primary ones being the decline in energy metabolism, the altered Ca^2+^ homeostasis and signaling, the decreases in synaptic neurotransmission, and the enhanced inflammatory responses. The genomic differences between vulnerable and resistant neurons fit quite well the hypothetical schemes already developed in the literature to account for the neuronal losses observed in aging and AD brains, i.e., the mitochondrial cascade hypothesis [125], the Ca^2+^ hypothesis of aging and AD [126, 127], the synaptic disruption hypothesis of AD and aging [128, 129], and the neuro-inflammation hypothesis of aging and AD [130]. 

Several of the functional categories of genes shown in Fig. (**2**), are also differentially expressed in neurons that are vulnerable to OS, regardless of their location in the brain [65, 66]. Included among these categories are those of enhanced immune/inflammatory response, decreased energy generation, reduced signal transduction, and dysregulation of Ca^2+^ signaling. The oxidative stress hypothesis of aging is one of the predominant theories of age-associated cell damage and death in many organs, including the brain [131]. Thus, the functional genomics approach to defining some of the molecular characteristics of neuronal vulnerability to aging and AD has confirmed most of the major hypotheses of molecular and cellular changes proposed as key processes leading to neurodegeneration in aging and AD. What the summary of the functional genomic studies of brain in aging and AD have offered beyond the identification of potential pathways to neuronal vulnerability are key genes and gene products related to these pathways and whose expression is intrinsically different in vulnerable vs. resistant neurons. The identification of specific genes in these pathways that may determine vulnerability of neurons can be used for future drug development to prevent the loss of neurons in neurodegenerative diseases such as AD. For example, the development of drugs against specific targets that control calcium signaling or against some of the inflammatory response factors may provide the means to protect selectively vulnerable neurons from further damage in AD. Mitochondrial metabolic dysfunction, either because of down-regulation of the expression of ETC enzymes or mitochondrial membrane translocases, such as TOMM40, may also become an attractive target for the development of agent that delay the stress of the aging process or the initiation and continuation of the damage induced in AD-sensitive neuronal populations. 

## Figures and Tables

**Fig. (1):**
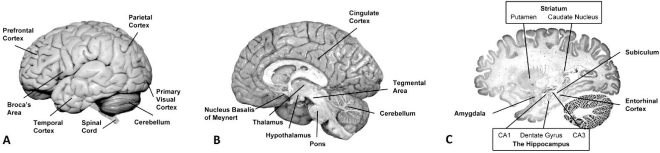
Human brain structures examined in this review. Different views are shown in three panels: (**A**) lateral view of the left hemisphere, (**B**) medial view of the right hemisphere, and (**C**) sagittal view of one hemisphere. (The image in Panel **C** is adapted with permission from http://www.brains.rad.msu.edu, supported by the US National Science Foundation).

**Fig. (2):**
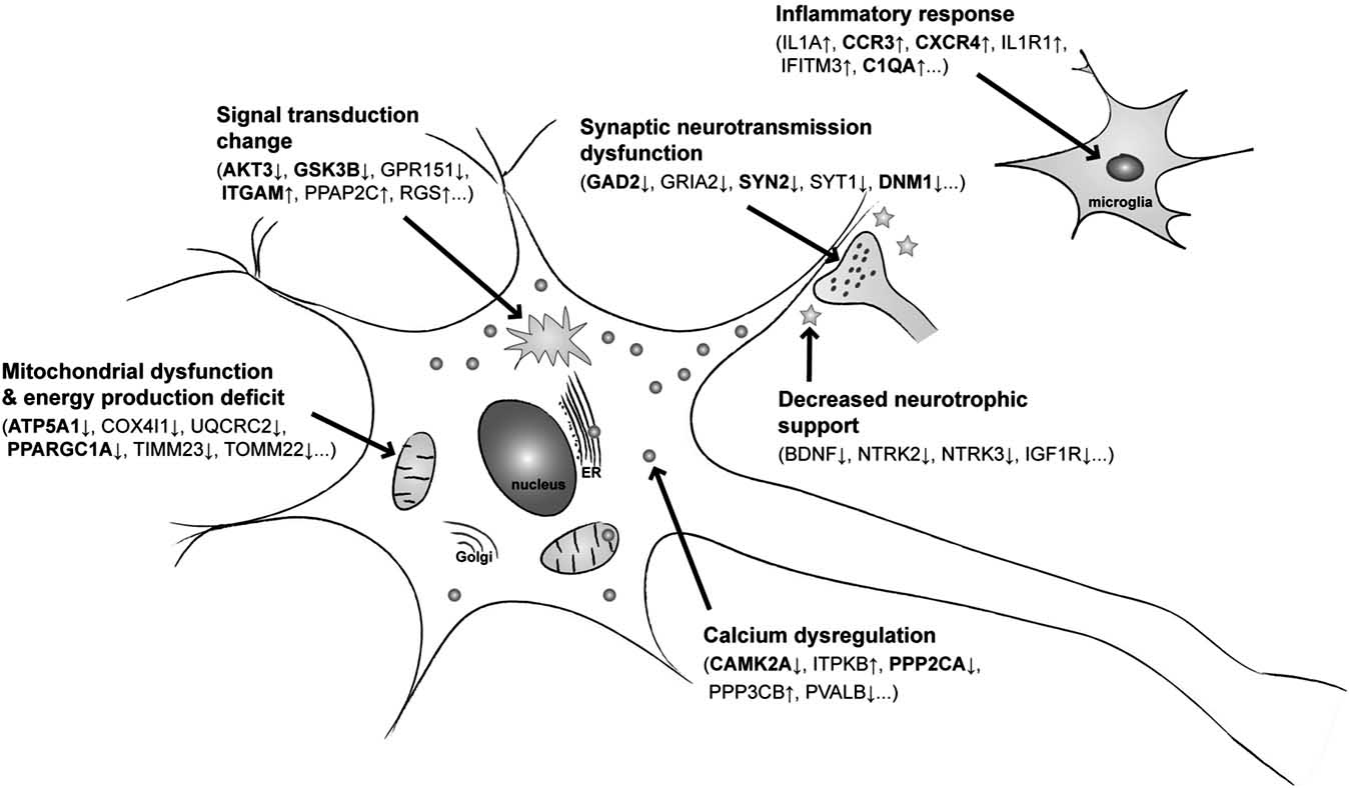
Major functional categories of genes whose expression is altered in neurons frequently damaged in AD. Genes in parentheses are some of the most representative and those that are most frequently detected as being over- or under-expressed in AD brains in more than one study (see Table **3**). The genes in boldface type are those for which the respective protein levels have been confirmed as ones that change in AD in the same direction as the gene expression levels. The arrows after each gene represent the direction of their respective change in vulnerable regions during AD pathogenesis. Some of the major gene expression patterns characteristic of vulnerable neurons in AD are also shared by neurons that are susceptible to the effects of the aging process, as well as neurons sensitive to the damaging effects of oxidative stress (see text).

**Table 1:** Transcriptomic Studies and Gene Categories that Characterize Selectively Vulnerable Regions in Human AD Brains

Sample source	Technology	Gene categories with altered expression in AD	Refs.
***I. Region-to-region comparative studies***			
Amygdala and cingulate cortex (vulnerable) *vs*. striatum and cerebellum (resistant)			
6 AD brains (83.0±2.6 [mean±SD] yrs; 3Female+3Male; CERAD* diagn.), 9 controls (65.9±13.5 yrs; 2F+7M)	Unigene Lifearray microarrays (InCyte)	Up-regulation of chronic inflammation and down-regulation of signal transduction and energy metabolism in vulnerable regions	[85]
Entorhinal cortex (vulnerable) vs. dentate gyrus (resistant)			
6 AD brains, 6 controls (Information not available)	GeneChip Human Genome HG-U133A	VPS35 and retromer complex	[132]
Entorhinal cortex (EC), hippocampus (HIP), middle temporal gyrus (MTG), and posterior cingulate cortex (PCC) (vulnerable) vs. superior frontal gyrus (SFG) and primary visual cortex (VCX) (resistant)			
33 AD brains (79.9±6.9 yrs; 18F+15M; Braak III-VI), 14 controls (79.8±9.1 yrs; 4F+10M) (neurons obtained by LCM)	GeneChip Human Genome U133 Plus 2.0	Synaptic transmission and synaptic vesicle transport down-regulated in EC and HIP. Microtubule-based movement and axon cargo transport decreased in MTG and PCC. Energy metabolism genes significantly reduced in all vulnerable regions compared to resistant regions	[71, 72]
Hippocampus (vulnerable) vs. parietal cortex (resistant)			
4 AD brains (81.5±7.1 yrs; 1F+3M; Braak III), 4 controls (87.8±4.7 yrs; 3F+1M; Braak I-II)	UniGem V array (Incyte)	Calcineurin Aβ up-regulated in vulnerable region	[123]
***II. Single vulnerable region ***			
**Cerebral cortex**			
Frontal cortex			
61 AD brains (78.0 yrs [mean]; 31F+30M), 23 controls (73.0 yrs, no gender information)	Two custom cDNA microarrays	Calcium dysregulation in AD	[73]
6 AD brains (85±7 yrs), 6 controls (74±10 yrs)	Atlas human 12K microarray (Clontech)	Increased production of interferon-γ and up-regulation of interferon-induced genes in AD brain	[94]
13 AD brains (82.8±6.2 yrs; 10F+3M), 11 controls (75.8±10.5 yrs; 5F+6M)	Affymetrix HuFL GeneChips	Down-regulation of synaptic vesicle trafficking in AD brains	[112]
Inferior parietal lobe			
10 AD (82.3±6.7 yrs; 4F+6M; Braak V-VI), 6 demented but non-AD (70.7±9.0 yrs; 2F+4M), 10 non-demented controls (84.9±5.4 yrs; 7F+3M; Braak≤3)	Agilent 44k microarray	Immune response, CNS development, and Aβ processing and disposition were among the most changed in AD brains	[96]
Superior temporal gyrus			
8 AD brains (72.6±9.1 yrs; 3F+5M; Braak V/VI (except 2 III/IV), 8 controls (80.1±7.1 yrs; 3F+5M; Braak 0-II (except 1 III/IV))	GeneChip Human Exon 1.0 ST Arrays	Synaptic dysfunction, perturbed neurotransmission, and activation of neuroinflammation in AD brains	[98]
5 AD brains (moderate dementia; CDR** 2), 6 controls (CDR 0)	UniGem V1 array (Incyte)	Synaptic vesicle protein synapsin a-type isoform selectively decreased in vulnerable regions of AD brains	[111]
**Hippocampus**			
Whole hippocampus			
2 AD brains (80,82 yrs; both M), 3 controls (58,68,78 yrs; all M)	GeneChip Human Genome HG-U133A	Genes related to energy metabolism were down-regulated in the AD-affected region	[88]
AD brains (83.2±8.1 yrs; 10F+8M; CDR 3-5) and controls (78.5±14.7 yrs; 6F+5M; CDR 0)	Genome-wide cDNA array	Impaired glucose/energy metabolism and attenuation of PGC-1α expression; hypothesized to lead to Aβ generation and amyloidogenesis	[89]
Hippocampal CA1			
22 AD brains (7 Incipient: 90.0±5.6 yrs, MMSE*** 20-26; 8 moderate: 83.4±3.1 yrs, MMSE 14-19; 7 severe: 84.0±10.6 yrs, MMSE<14), 9 controls (85.3±8.1 yrs; MMSE>25)	GeneChip Human Genome HG-U133A	Up-regulation of tumor suppressors, oligodendrocyte growth factors, protein kinase A modulators, cell adhesion, apoptosis, lipid metabolism, and inflammation; down-regulation of protein folding/metabolism/transport, energy metabolism and signaling in AD brains	[90]
6 AD brains (70.3±3.3 yrs; 3F+3M; CDR 2-3), 6 controls (69.0±1.8 yrs; 3F+3M; CDR 0)	GeneChip Human Genome U95Av2	Dysregulation of metal ion homeostasis; down-regulation of transcription factor signaling and neurotrophic support; upregulation of apoptotic and neuroinflammatory signaling in AD	[97]
5 AD brains (77.2±7.4 yrs; 2F+3M), 5 controls (73.2±9.8 yrs; 2F+3M) (CA1 neurons obtained by LCM)	Incyte GDA array and custom cDNA array	CA1 neurons with neurofibrillary tangles have significant reductions in phosphatases/kinases, cytoskeletal proteins, synaptic proteins, glutamate receptors, and dopamine receptors in AD	[70]
**Nucleus basalis of Meynert**			
10 AD brains (84.5±4.0 yrs; 7F+3M; MMSE 14.8±8.6), 6 controls (81.5±7.2 yrs; 4F+2M; MMSE 27.4±1.5) (cholinergic neurons collected by single-cell aspiration)	Custom cDNA arrays	Neurotrophin receptors, synaptic proteins and protein phosphatases down-regulated in AD brain	[113]

* CERAD: Consortium to Establish a Registry for Alzheimer's Disease;

** CDR: clinical demention rating;

*** MMSE: Mini-Mental State Examination.

**Table 2:** Transcriptomic Studies that Characterize Selective Vulnerability in Brains from Mouse Models of AD

Sample source	Technology	Gene categories with altered expression in AD	Refs.
***I. Region-to-region comparisons***			
Hippocampus and cortex (vulnerable) *vs*. cerebellum, striatum and brainstem (resistant)			
4 APP+PS1 transgenic, 4 non-transgenic controls (17-18 mos littermates)	Rat LifeArray 1 & 2 (InCyte)	Down-regulated memory consolidation and up-regulated inflammatory genes in transgenic mice	[93]
***II. Single vulnerable region***			
**Cerebral cortex**			
Whole cerebral cortex			
10 Balb/c with icv inj. of aggregated Aβ25-35, 10 with saline (weight 22-24 g)	Atlas Mouse 1.2 Expression Array	Apoptosis, energy metabolism, calcium ion homeostasis dysfunction, cell adhesion and neuronal dystrophy in Aβ-treated mice	[86]
3 AD model genotypes: APP/PS-1^P264L/P264L^(4 & 18 mos, n=3 each age), Tg2576/PS-1^P264L/P264L^ & Tg2576/PS-1^P264L/+^(2 & 12 mos, n=3 each age/genotype) and wild-type mice (n=3 corresponding to each genotype)	GeneChip Murine Genome U74A	Up-regulated in the 3 AD models: immune response, carbohydrate metabolism, and proteolysis. Down-regulated: pituitary adenylate cyclase-activating peptide (PACAP), brain-derived neurotrophic factor (BDNF), and insulin-like growth factor I receptor (IGF-IR)	[87]
Frontal cortex			
15 PS1/PS2 double knockout mice and 15 wild-type controls (2, 4, 6, 7 & 8 mos; 3 animals pooled at each age)	GeneChip Mouse Genome 430	Progressive, age-dependent up-regulation of neuroinflammation and cathepsin in transgenic mice	[95]
**Hippocampus**			
Whole hippocampus			
5 APPsw transgenic mouse brains, 5 non-transgenic controls (12 mos littermates)	ABI mouse genome survey array	Up-regulated: signal transduction and protein binding; down-regulated: extracellular space, protein binding, cell communication, and transporter activity in transgenic mice	[124]
15 PS1/PS2 double knockout mice and 15 wild-type controls (2, 4, 6, 7 & 8 mos; 3 animals pooled at each age)	GeneChip Mouse Genome 430	Progressive, age-dependent up-regulation of neuroinflammation and cathepsin in transgenic mice	[95]

**Table 3:** Functionally Categorized Key Genes Identified in Transcriptomic Studies as Being Characteristic of Vulnerable Regions in Human AD or Animal Models of AD (Underlined: Down-Regulated Genes; Non-Underlined: Up-Regulated Genes)

Genes	Selection Criteria	Protein confirmation	Species	Study
*Mitochondrial metabolism and energy production*				
COX4I1, TPI1, TPI1P1, LMO4, NDUFS5, LOC100130794, LOC652797, PKM2, ENO3, UQCRC2, OXCT1, SLC25A4	FC* > 2.0	nc**	Human	[85]
OXPHOS: ATP5A1, ATP5B, ATP5C1, ATP5G3, ATP5O, COX5B, NDUFA1, NDUFA10, NDUFA11, NDUFA13, NDUFB8, SDHB, UQCRC1, UQCRC2, etc. Mito membrane translocases: TIMM17A, TIMM23, TIMM50, TOMM22, TOMM34	P < 0.01 (FDR***)	Subunits of Complex I-V	Human	[72]
Glycolysis: GAPD, GPI, LDHA; TCA: ACLY, FH, MDH1, MDH2, SUCLA2; OXPHOS: ATP5B,COX4I1, COX6A1, NDUFS4, UQCRC2, SLC25A14	P < 0.05	nc	Human	[88]
Glycolysis: GAPD, GPI, PFKM, PGK1; TCA: ACLY, CS; OXPHOS: COX6C, MTATP6; Pyruvate metabolism: PC, PCK2, PDHA1, PDK3; Others: PPARGC1A (PGC-1α), UCP2	P ≤ 0.01	PPARGC1A (PGC-1α)	Human	[89]
ACAD8, ALDH2, COX7A2L, COX7C, HADH, NDUFB3, PDHA1, PDHX, SDHA,SLC25A6	P < 0.05 (FDR)	nc	Human	[90]
Carbohydrate metabolism: FUCA1, GUSB, MAN2B1		nc	Mouse	[87]
*Neuroinflammatory response*				
C3, HLA-DRB5, CD74, CD99	FC > 2.0	nc	Human	[85]
IFITM3	FC > 5.0	nc	Human	[94]
CCL5, C4A, IL1F7, IL1R1, NFKB2, TGFB1, TGFB3, TNFSF7	FDR ≤ 10%		Human	[98]
CCL27, CCR3, CCR5, CXCR2 (IL-8Rβ), CXCR4, IL-28A	P < 0.02 (Permutation)	CCR3, CXCR4	Human	[96]
IFNA5, IFNG, IL10RA, IL10RB, IL13RA1, IL17R, IL18, IL2RG, IL6R	P < 0.05 (FDR)	nc	Human	[90]
APP, B94, CEX1, DPP1, HB15, HUMJE, IFNIND, IL1A (IL-1α), IL1B (IL-1β), NF-IL6, NFKBIA, PLA2G4A, PTGS2, RELB	FC ≥ 3.0& P ≤ 0.05	nc	Human	[97]
C1QA, C1QB, C4A, CBS, THRA, GFAP	FC > 1.4& P < 0.05	nc	Mouse	[93]
AKT1, CSF2RB2, GPI1	FC ≥ 2.0	nc	Mouse	[86]
C1QA, C4A, CD14, FCGR1, GFAP, IGH-6	FC ≥ 1.2& P ≤ 0.05	nc	Mouse	[87]
C1QA, C1QB, C1QC, C3AR1, C4B	FC > 2.0& P < 0.05	C1QA	Mouse	[95]
*Synaptic transmission and synaptic vesicle transport*				
CHRNB2, DRD1IP, GABRA1, GABRA4, GABRG2, GAD1, GAD2, GRIA2, GRIA3, GRIA4, GRIK1, GRIK4, GRINA, GRIN2A, GRIN2D, GRM4, HTR1B, HTR2A, HTR3B, NRN1, SNAP25, STX12, SV2A, SV2B, SYNJ1, SYT8, SYT11, SYT12, SYT13, VAMP1	FDR ≤ 10%	GAD65 (GAD2)	Human	[98]
DNM1, STX1A, SYT1	P < 0.05	DNM1	Human	[112]
SYN2(a)	FC ≥ 1.8	Synapsin Ia, IIa, IIIa	Human	[111]
CHAT, CSPG5, NFL, SLIT2, SYN1, SYP	FC ≥ 3.0& P ≤ 0.05	nc	Human	[97]
ARC, SLC6A3 (DAT), DRD1, DRD2, DRD3, DRD4, DRD5, GRIA1, GRIA2, GRIN2B, SYN1, SYP, SNCA, SNCB, SYTx	FC ≥ 2.0	nc	Human	[70]
SYP, SYTx	P ≤ 0.05	nc	Human	[113]
ARC, ATP1A3, EGR1 (Zif268), NR4A1 (Nur77/TR3)	FC > 1.4& P < 0.05	nc	Mouse	[93]
*Calcium regulation*				
CAMK2A, CAMK2G, ITPKB, PPP2CA, PPP3CA, PVALB	FDR ≤ 10%	CAMKII, PP2AC (PPP2CA)	Human	[98]
PPP3CB (Calcineurin Aβ)	FC>2.0	nc	Human	[123]
ITPKB, RGS4	P < 0.05	nc	Human	[73]
CAST, DAPK2, EP300, S100A4	P < 0.05 (FDR)	nc	Human	[90]
*Cytoskeletal function*				
VPS35	P < 0.01	VPS35	Human	[132]
NF-L, NF-M, NF-H, TUBB	FC ≥ 2.0	nc	Human	[70]
*Signal transduction*				
AHNAK, CHL1, COPS8, ENSA, FKBP4, GSK3A, ITPR1, MAPK10, MAP2K1, MAP3K6, NOMO3,NOMO1,NOMO2, NPTN, NR4A1, PGRMC1, PIP4K2B, PPAP2C, PPP2CA, PRKACB, PRKCZ, PTK2, RAN, RCAN2, RGS5, RUNDC3A, SIRPA,YWHAB	FC > 2.0	nc	Human	[85]
AIM1, CDK2AP1, FZR1, GSK3B, SFRP1, TGFBR3	P < 0.05 (FDR)	nc	Human	[90]
AKT3, CDK5, GNA12, GNB5, GNG5, GSK3B, MAPK4, MAPK6, MAPK8, MAPK9, PLCB2, PPP3CB, PPP2R1B, PRKCB1, PRKCD, PRKCE, PRKCZ, RGS4	FDR ≤ 10%	AKT3, GSK3β	Human	[98]
ADAM9, CDP5, DAXX, FAS	FC ≥ 3.0& P ≤ 0.05	nc	Human	[97]
ERK1, ERK2, FAK, GSK3B, NOS2, PPP1CA, PPP1CC, PPP2R1A, PPP2CA	FC ≥ 2.0	nc	Human	[70]
PPP1CA, PPP1CC	P ≤ 0.05	nc	Human	[113]
AKT1, CDH2, ERBB2, FGFR1, FZD3, FZD7, ITGAL, ITGAM, JUN, LIMK1	FC ≥ 2.0	ITGAM	Mouse	[86]
ADORA2A, ATP2A1, BMP2, CACNG8, CAMTA2, CAP1, CIDEA, DUSP9, GAL, GNG8, GPR27, GPR77, GPR151, HCN2, KCNA5, MAP3K8, OXT, PDE6G, PLA1A, PRKAG3, RBL1, S100A8, SHC1, SMAD4	FC ≥ 2.0& P < 0.05	nc	Mouse	[124]
*Protein folding and proteolytic activity*				
AHSA1, CCT2, CCT3,CCT4, CCT5, DNAJA1, HSP90AB1, HSP90AA2, HSP90AA1, PDIA6	P < 0.05 (FDR)	nc	Human	[90]
CTSD	FC ≥ 2.0	nc	Human	[70]
CTSD	P ≤ 0.05	nc	Human	[113]
CTSD, CTSH, CTSS	FC ≥ 1.2& P ≤ 0.05	nc	Mouse	[87]
CTSC, CTSD, CTSH, CTSS, CTSZ	FC > 2.0& P < 0.05	CTSD	Mouse	[95]
*Neurotrophic support*				
BDNF, HER2B, P2XR	FC ≥ 3.0& P ≤ 0.05	nc	Human	[97]
NTRK2 (trkB), NTRK3 (trkC)	P ≤ 0.05	nc	Human	[113]
ADCYAP1 (PACAP), BDNF, IGF1R	FC ≥ 1.2& P ≤ 0.05	nc	Mouse	[87]

* FC: Fold Change;

** nc: no confirmation;

*** FDR: Benjamini and Hochberg False Discovery Rate.

## ABBREVIATIONS

Aβ: = Amyloid β peptide
AD: = Alzheimer’s disease
APP: = Amyloid precursor protein
CDR: = Clinical demention rating
CERAD: = Consortium to Establish a Registry for Alzheimer's Disease
CMR_GI_: = Cerebral metabolic rate of glucose
CNS: = Central nervous system
ETC: = Electron transport chain
FC: = Fold change
FDR: = Benjamini and Hochberg false discovery rate
LCM: = Laser capture microdissection
MMSE: = Mini-Mental State Examination
nc: = No confirmation
NFT: = Neurofibrillary tangle
OS: = Oxidative stress
PET: = Positron emission tomography
PS1: = Presenilin 1
ROS: = Reactive oxygen species
SNV: = Selective neuronal vulnerability
